# Early Diagnosis and Surgical Management of Boerhaave Syndrome: A Case Report

**DOI:** 10.7759/cureus.47596

**Published:** 2023-10-24

**Authors:** Albion Totsi, Konstantinos Fortounis, Stamatia Michailidou, Nikolaos Balasas, Christos Papavasiliou

**Affiliations:** 1 Surgical Department, Papageorgiou General Hospital, Thessaloniki, GRC

**Keywords:** early diagnosis, surgical management, mediastinitis, esophageal perforation, boerhaave syndrome

## Abstract

Boerhaave syndrome is a rare condition of spontaneous esophageal perforation after multiple episodes of forceful emesis. Due to its high morbidity and mortality rates, early diagnosis and treatment are key prognostic factors. Herein, we present a case of Boerhaave syndrome, which was initially misinterpreted as a coronary event due to similar confusing symptoms. However, a diagnosis was made without delay and confirmed with a chest computed tomography (CT) scan, which revealed pneumomediastinum. The patient was treated surgically by primarily repairing the rupture with an omentum patch reinforcement, draining the mediastinum and both pleural cavities, and creating a feeding jejunostomy. After a long stay in the ICU and the Surgical Department, the patient was discharged in good clinical condition with normal oral feeding.

## Introduction

Esophageal perforation is a serious condition that, if not treated in time, leads to high mortality rates [1]. The most common cause is iatrogenic, followed by other causes such as trauma, esophageal tumors, foreign bodies, and spontaneous perforation. The last is responsible for about 15% of esophageal perforations, and it is a condition known as Boerhaave syndrome, which is due to multiple episodes of forceful emesis [2]. The first description was made in 1724 by Herman Boerhaave, who analyzed the spontaneous esophageal perforation of the Grand Admiral of the Dutch fleet [3].

The main symptom of patients with Βoerhaave syndrome is chest pain that follows episodes of forceful vomiting with clinical examination revealing a subcutaneous emphysema. This clinical manifestation of the disease is known as Meckler's triad, which is present in only 14% of patients. Other symptoms include dyspnea and pain in the back or neck. As mediastinitis tends to develop quickly after contamination of the mediastinum, systemic manifestations such as fever, tachycardia, and hypotension can also be present in cases of late admission to the hospital [4,5].

The indicated image examination for the diagnosis of esophageal perforation is contrast-enhanced computed tomography (CT) and CT esophagography, with a sensitivity of 92-100% [6]. CT scans not only assess the location of the perforation and the extent of mediastinal and chest contamination, but they also help us to rule out other conditions that can present with similar symptoms [6,7].

Management of Boerhaave syndrome can be divided into three columns: conservative strategies, early surgical repair, and endoscopic methods. Regardless of the treatment option, what is of utmost importance in esophageal perforation is the control of mediastinitis with appropriate drainage and antibiotic agents in combination with sufficient nourishment for the patient [8].

Here, we present a case of Boerhaave syndrome, which was initially mistakenly managed as a cardiac event. Nevertheless, early recognition of the condition and immediate surgical treatment were decisive for the final outcome. They are discussed below with a review of relevant literature.

## Case presentation

**Figure 1:**
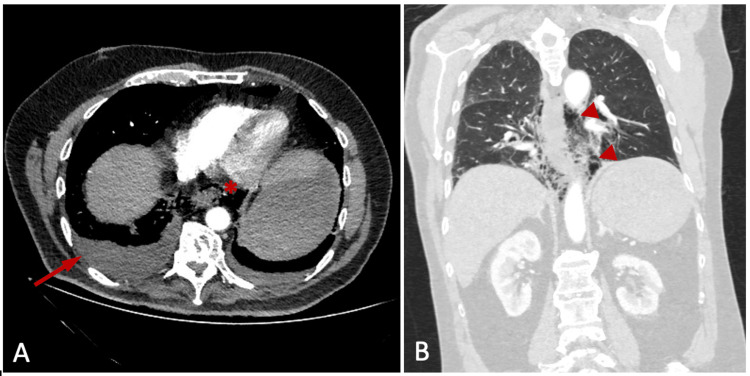
Chest CT scan findings in Boerhaave syndrome. (A) Axial plane which shows the free mediastinal air (*) and right pleural effusion (arrow); (B) coronal plane showing the extent of pneumomediastinum (arrowhead).

A 70-year-old man presented to the emergency department after midnight complaining of sudden and acute chest pain. He also exhibited tachycardia and shortness of breath and reported five forceful episodes of vomiting preceding the pain. His past medical history included hypertension, diabetes mellitus, and coronary artery disease, for which he had undergone a percutaneous coronary intervention (PCI) two years earlier. The history of coronary artery disease led clinicians to suspect an acute coronary event, and consequently, the patient was initially admitted to the coronary care unit. On physical examination, his abdomen was tender in the epigastrium, whereas palpation and auscultation of his thorax did not reveal any abnormal findings. The chest X-ray, which was performed with the patient in a supine position, did not show any pathology. Laboratory tests revealed elevated leukocytes at 16.60 K/μl (normal 3.70-9.50) with 84.9% neutrophils. As cardiac enzymes and electrocardiograms were normal, the patient was further investigated with chest computed tomography, which revealed a bilateral pleural effusion with the largest amount on the right and free mediastinal air located next to the distal third of the esophagus (Figure 1). Therefore, the diagnosis of esophageal perforation and Boerhaave syndrome was confirmed.

**Figure 2:**
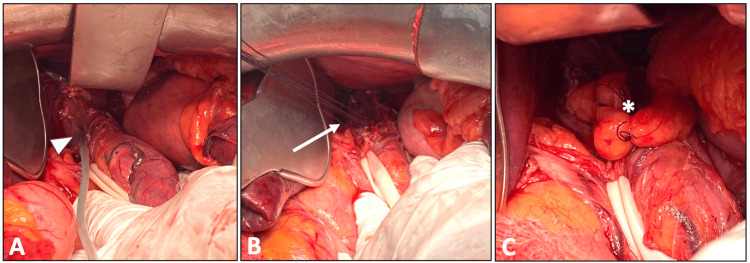
Intraoperative pictures of esophageal rupture repair. (A) Pediatric feeding tube placed into the esophageal rupture (arrowhead); (B) defect repair with individual sutures (arrow); (C) reinforcement of repair with an omentum patch (*).

The patient was immediately transferred to the operating theater, and an explorative laparotomy was performed. After the mobilization of the abdominal and lower thoracic esophagus, the exact location of the rupture was identified, approximately 1 cm above the cardioesophageal junction. The defect was repaired using individual sutures made of 4-0 polydioxanone and reinforced with an omentum patch, whereas transabdominal drainage was placed in the mediastinum (Figure 2). Furthermore, a feeding jejunostomy was performed, and a thoracic tube was left in each pleural cavity. The patient was transferred to the intensive care unit (ICU), hemodynamically unstable with lactic acidosis and a need for vasopressors.

The initial management in the ICU was focused on the treatment of sepsis and mediastinitis with antibacterial, antifungal, and proton-pump inhibitor medications, whereas nutrition was ensured through feeding jejunostomies. In the first days, the patient remained in septic shock with a fever and high inflammatory markers. Gradually, his general condition improved, and he was removed from mechanical ventilation. During his stay in the ICU, a contrast chest and abdomen computed tomography was performed, giving gastrografin through the nasogastric tube. The CT scan did not reveal esophageal leakage or pleural or mediastinal effusions. Therefore, chest tubes and abdominal drainage were removed.

After 21 days in the ICU, the patient returned to the surgical department in a stable condition. Before starting oral feeding, the patient underwent an esophagram with gastrografin, which did not show any leak but a normal liquid transit from the esophagus to the stomach and thereafter to the duodenum. Therefore, he started progressively eating soft foods without any complications and also showed improvement in his mobilization. His hospitalization was complicated by a urinary tract infection, which was treated with targeted antibiotic medication. Eventually, he was discharged on the 50th postoperative day in generally good condition. One month later, as part of the follow-up, he visited our department and underwent a new chest and abdomen CT scan, which did not reveal any notable pathological findings. Two months after his discharge, and given that his oral feeding was normal, the feeding jejunostomy was removed.

## Discussion

The pathogenic mechanism of Boerhaave syndrome, which leads to the transmural tear of the esophagus, is that of a rapid increase in intraesophageal pressure combined with negative intrathoracic pressure during episodes of vomiting. During this procedure, the function of the lower esophageal sphincter is normal [4]. The most frequent and weak site where the rupture of the esophagus occurs is in its lower third on the left side and just above the diaphragm, while the direction of the rupture is longitudinal. The other parts of the esophagus are rarely subject to rupture [9].

Due to its rarity, Boerhaave syndrome can easily be confused with other more common conditions with a similar clinical presentation. The differential diagnosis includes diseases of the thoracic region such as myocardial infarction, pneumothorax, pericarditis, pulmonary embolism, and pathologies of the abdominal cavity such as perforated peptic ulcer and pancreatitis [4]. In our case, the normal cardiac enzymes in combination with the history of vomiting episodes helped us further investigate the possibility of esophageal perforation. A chest CT scan was the appropriate imaging method, which confirmed the diagnosis and evaluated the magnitude of the contamination.

The time of patient presentation to the hospital plays a crucial role in the management of esophageal perforation. When treatment begins 24 hours after the onset of symptoms, the prognosis of patients improves significantly (mortality rates range from 30% to less than 10%). In some cases that meet specific criteria, conservative management can be feasible. Some such criteria are an early presentation, hemodynamic stability, a contained esophageal rupture, and limited mediastinal contamination [6]. The next, less invasive choice of treatment that has gained increased popularity in the last decade is endoscopic management, which has a variety of options in its arsenal, such as covered metal stents, endoscopic clips, or endoluminal vacuum therapy [10]. For patients who are not candidates for either endoscopic or non-operative management, surgery (open, laparoscopic, or thoracoscopic) tends to provide the solution, especially in unstable cases with signs of significant mediastinitis. Surgical treatment aims to find and close the esophageal defect, when possible, drain the mediastinum and pleural cavities adequately, and ensure a safe feeding route for the patient. If primary repair is unattainable, exclusion and diversion, esophagectomy, or drainage alone can be alternative solutions [6,11].

Our patient was taken into the operating theater less than 24 hours from the onset of the symptoms in a stable clinical condition. The findings of the CT scan, which revealed extensive contamination of the mediastinum and the right pleural cavity, were decisive for our choice of surgical treatment. As the location of the rupture was suspected to be in the lower esophagus, near the esophagogastric junction, the transabdominal approach was considered to be the ideal option. In this way, complications and higher morbidity rates of thoracotomy are prevented. After pinpointing the exact location of the defect, it was necessary to perform debridement of non-viable tissue before suturing in a single layer. We reinforced closure with a small tension-free omentum patch and drained all thoracic cavities. A last but important step was the construction of a feeding jejunostomy, which would ensure the patient’s nourishment during the catabolic phase of sepsis and help in esophageal healing.

After interventions for the esophageal rupture, the part of management that follows is the most critical for the patient’s prognosis and has to do with the control of the mediastinitis and the recovery from septic shock. Therapy starts with intravenous broad-spectrum antibiotics covering the aerobes and anaerobes bacteria that colonize the upper gastrointestinal tract until the results of cultures from tissue or fluid samples taken intraoperatively are ready. In some cases, the use of anti-fungal medication is also recommended [12,13]. The intensive care unit was responsible, in our case, for the management of the postoperative septic condition. The adequate drainage of the mediastinum and both pleural cavities also played a crucial role in controlling the mediastinitis.

## Conclusions

Due to its rarity and severity, early diagnosis of Βoerhaave syndrome is the first key to a better prognosis. Immediate surgical treatment with primary repair of the rupture, drainage of the mediastinum and pleural cavities, and the creation of a feeding route constitute a safe and effective approach to managing esophageal perforation. Especially when treatment is given less than 24 hours after the onset of the symptoms, the prognosis improves significantly.
